# Postnatal Zika virus infection leads to morphological and cellular alterations within the neurogenic niche

**DOI:** 10.1242/dmm.050375

**Published:** 2024-02-28

**Authors:** Jéssica C. C. G. Ferreira, Raissa R. Christoff, Tailene Rabello, Raiane O. Ferreira, Carolina Batista, Pedro Junior Pinheiro Mourão, Átila D. Rossi, Luiza M. Higa, Maria Bellio, Amilcar Tanuri, Patricia P. Garcez

**Affiliations:** ^1^ Institute of Biomedical Sciences, Federal University of Rio de Janeiro, Rio de Janeiro, RJ 21941-590, Brazil; ^2^ Department of Genetics, Institute of Biology, Federal University of Rio de Janeiro, Rio de Janeiro, RJ 21941-902, Brazil; ^3^ Microbiology Institute Paulo de Góes, Federal University of Rio de Janeiro, Rio de Janeiro, RJ 21941-902, Brazil

**Keywords:** Brain development, Dentate gyrus, Congenital malformation, Congenital Zika syndrome, Hippocampus, Microcephaly, Neurogenesis, Subgranular zone, Zika virus

## Abstract

The Zika virus received significant attention in 2016, following a declaration by the World Health Organization of an epidemic in the Americas, in which infections were associated with microcephaly. Indeed, prenatal Zika virus infection is detrimental to fetal neural stem cells and can cause premature cell loss and neurodevelopmental abnormalities in newborn infants, collectively described as congenital Zika syndrome. Contrastingly, much less is known about how neonatal infection affects the development of the newborn nervous system. Here, we investigated the development of the dentate gyrus of wild-type mice following intracranial injection of the virus at birth (postnatal day 0). Through this approach, we found that Zika virus infection affected the development of neurogenic regions within the dentate gyrus and caused reactive gliosis, cell death and a decrease in cell proliferation. Such infection also altered volumetric features of the postnatal dentate gyrus. Thus, we found that Zika virus exposure to newborn mice is detrimental to the subgranular zone of the dentate gyrus. These observations offer insight into the cellular mechanisms that underlie the neurological features of congenital Zika syndrome in children.

## Teaser

**Summary:** Following intracranial Zika virus infection in postnatal mice, the formation of the neurogenic niche within the dentate gyrus is affected and subgranular zone development is severely impaired.

## INTRODUCTION

The Zika virus (ZIKV) is of great interest in neuroscience, given its capacity to infect and kill human neural stem cells (NSCs) (Garcez et al., 2017, 2016; Tang et al., 2016). This cytotoxic trait is linked to its causative effects on congenital brain abnormalities, including microcephaly, ventriculomegaly and brain calcification. As described by the World Health Organization (WHO), such brain malformations that accompany ZIKV infection are collectively described as congenital Zika syndrome (CZS) (França et al., 2016; World Health Organization, 2016). In addition to these structural brain alterations, a 5-year follow-up study of cases following an outbreak in Brazil described additional neurological sequelae, including cerebral palsy, epilepsy and motor impairment (Nielsen-Saines et al., 2019; Pessoa et al., 2018).

To date, several experimental models that explore the neurodevelopmental impact of ZIKV infection, induced prenatally as well as postnatally, have been reported (Cugola et al., 2016; Garcez et al., 2018; Li et al., 2016; Nguyen et al., 2017). These studies show that, for *in vivo* analyses, the choice of experimental ZIKV infection is influenced by the age, the immunological status of the host, as well as the route of infection and the strain of virus. For example, animals infected intracerebrally have been documented to succumb to higher levels of infection compared with animals infected through intraperitoneal injection of virus (Dick et al., 1952). In another scenario, immunocompromised mice were found to be highly susceptible to ZIKV at any age (Lazear et al., 2016).

ZIKV has tropism for neural progenitors and plays a neurotoxic role, resulting in disruptions to many aspects of human brain development (Vhp et al., 2020). These cellular effects largely explain the brain alterations associated with early clinical findings related to CZS, such as microcephaly, ventriculomegaly and brain calcifications, and underpin subsequent neurological impairment. Correspondingly, such brain alterations are also described in studies with animal CZS models. For example, Cui et al. (2017) showed that mice infected with ZIKV during the embryonic period exhibited microcephaly and subsequently developed motor deficits and hind limb paralysis. Another study found that, following postnatal viral exposure, infected mice exhibited spontaneous seizures during the juvenile period, presented with learning deficits and were more susceptible to induced seizures as adults (Nem de Oliveira Souza et al., 2018). Additionally, studies in primates have shown that postnatal infections lead to long-term behavioral, motor and cognitive changes, which include the increase of emotional activity, decrease in social contact, balance loss and visual recognition memory deficits (Raper et al., 2020). Together, these studies have been invaluable in documenting the causal impact of ZIKV infection on brain development, as well as on features of the adult central nervous system (Figueiredo et al., 2019; Li et al., 2016; Vhp et al., 2020).

Despite the current progress, ZIKV infection in neonates is less well understood. Although a strong correlation between ZIKV infection and brain anomalies has been documented in the offspring of infected mothers (Mlakar et al., 2016), the description of CZS is relatively recent, and longitudinal studies of significant timescales to offer a complete picture of the disease mechanism are yet to be completed. There are many preclinical studies demonstrating that ZIKV infection during embryonic development disrupts brain formation through its effects on progenitor cells. However, the impact of postnatal infection on neurodevelopment, particularly in neurogenic regions of the cerebral cortex, such as the subventricular zone (SVZ) of the cerebral cortex and the subgranular zone (SGZ) of the hippocampal dentate gyrus (DG), is less well understood. In this study, we explored the effects of postnatal ZIKV infection on the formation of the DG in a murine model. Our findings have implications for the clinical impact of neonatal ZIKV exposure on postnatal neurogenesis, as well as learning and memory in the young.

## RESULTS

### Postnatal ZIKV infection leads to morphological alterations in the hippocampal DG in a murine model

Within the mammalian cerebral cortex, the SGZ of the hippocampal DG has the capacity to generate new postnatal neurons that can integrate into the hippocampal circuitry, which subsequently perform important roles as functional circuits that underpin memory formation and learning (Altman, 1962; Altman and Bayer, 1990). In mice, this brain region begins to form at postnatal day (P) 0, when resident NSCs have migrated from their birth locations in the embryonic day (E) 13.5 medial pallium neuroepithelium to the nascent P0 DG (Morales and Mira, 2019). Next, the NSCs develop to establish a putative SGZ by P7, but this area is not clearly delineated until P14 (Nicola et al., 2015). Given its cytotoxic effects on NSCs in embryonic cortical development (Cugola et al., 2016; Garcez et al., 2018; Li et al., 2016; Nguyen et al., 2017), we explored the impact of neonatal ZIKV infection on NSCs of P0 mice as follows.

**Fig. 1.**
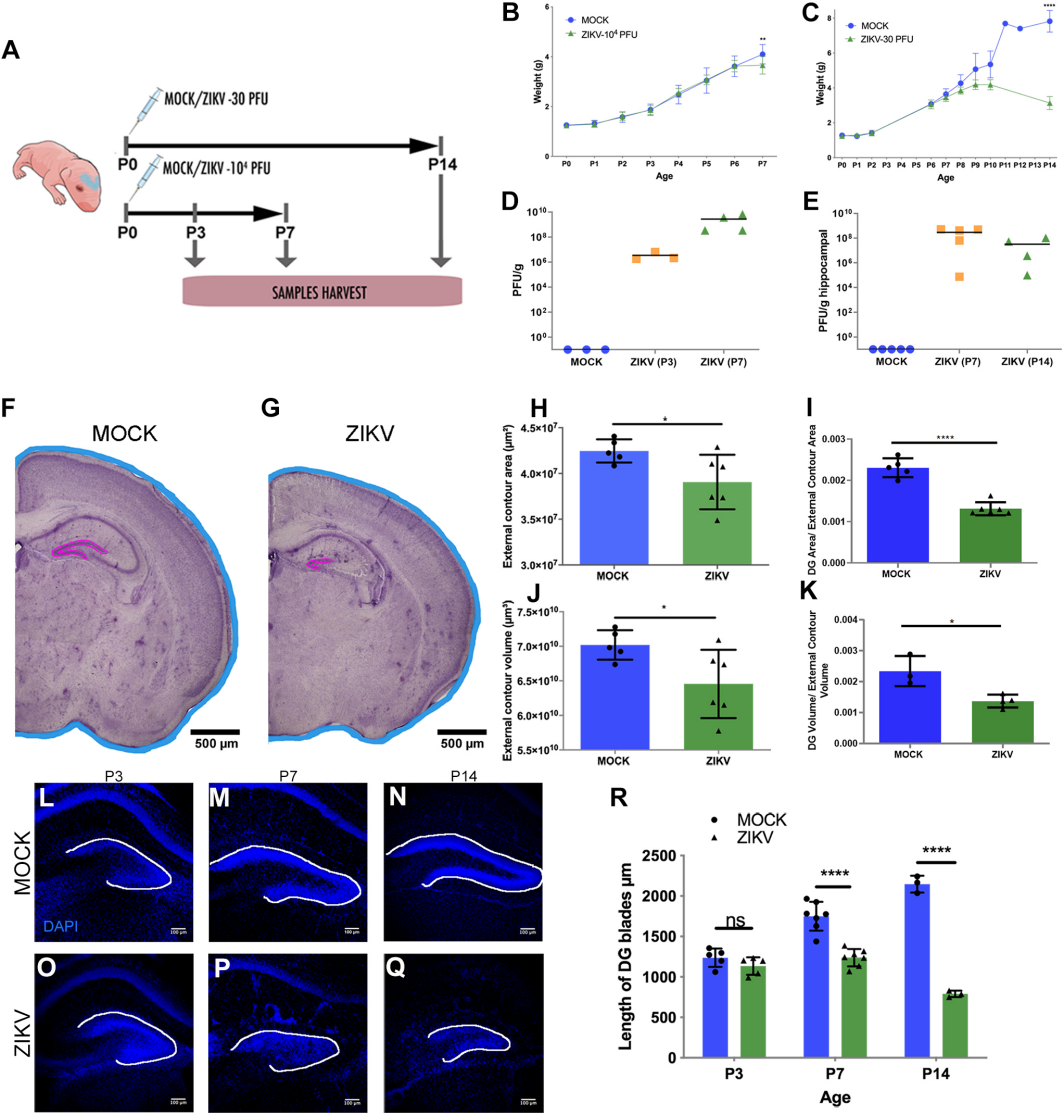
**Postnatal ZIKV infection leads to morphological alterations in the DG in a murine model.** (A) Illustration of the postnatal infection model. Mice were injected intracerebroventricularly at postnatal day (P) 0 with either mock solution or 10^4^ plaque forming units (PFUs) of ZIKV-BR or ZIKV-AF, and samples were collected at P3 and P7. For the animals that had samples harvested at P14, the injection was milder, with only 30 PFU of ZIKV-BR injected. Image created with mindthegraph.com. (B) Weight gain (g) in the first 7 days following 1×10^4^ PFU intracranial injection of ZIKV-BR. *n*=20 mock-treated and 20 ZIKV-infected animals from six different litters. **P<0.005. (C) Weight gain (g) in the first 14 days post 30 PFU intracranial injection of ZIKV-BR. ****P<0.00005. (D) Viral load measured by plaque brain assay (in PFU/g). *n*=10 (3 mock-treated animals, 3 animals injected with 10^4^ PFU ZIKV-BR and analyzed at P3, and 4 animals injected with 10^4^ PFU ZIKV-BR and analyzed at P7). (E) Viral load measured by plaque hippocampal assay (in PFU/g). *n*=14 (5 mock-treated animals, 5 animals injected with 10^4^ PFU ZIKV-BR and analyzed at P7, and 4 animals injected with 30 PFU ZIKV-BR and analyzed at P14). (F,G) Representative images of the forebrain coronal hemisections of mice injected with mock solution or 10^4^ PFU of ZIKV-BR stained with Cresyl Violet at P7 for morphometric analysis. Blue lines show the limits of the forebrain area in the sections (external contours) and the pink lines show the dentate gyrus (DG). Scale bar: 500 μm. (H-K) Quantification of the external contour area and volume (H,J) and quantification of the DG area divided by the external contour area and volume (I,K). *n*=11 (5 mock-treated and 6 ZIKV-injected animals). *P<0.05; ****P<0.00001. (L-Q) Representative images of coronal sections of the DG at 3, 7 and 14 days post injection with mock solution or 10^4^ PFU of ZIKV-BR stained with DAPI. The white lines show the lengths of the DG blades. Scale bar: 100 μm. (R) Quantification of the lengths of the DG blades. *n*=30 (15 mock-treated and 15 ZIKV-injected animals). ns, not significant; ****P<0.00001. All data are presented as mean±s.e.m. Unpaired two-tailed Student's *t*-test was used for statistical analysis.

We used a murine model of postnatal infection in newborn mice (P0), which correlates with an infection in the third gestational trimester in humans (Workman et al., 2013). In this approach, wild-type C57BL/6 mice were infected through bilateral, intracranial injections of a solution comprising 10^4^ plaque-forming units (PFUs) of Brazilian ZIKV (ZIKV-BR) per injection (Fig. 1A). The mock group received bilateral intracranial injections of vehicle only. To confirm viral presence, we analyzed the brain tissue and, more specifically, the hippocampus by the plaque assay (Fig. 1D,E). The weights of animals from each group were measured each day up to P7 (Fig. 1B) and we found that ZIKV-BR-infected mice had a significantly different weight by P7. In parallel, we studied a group of animals injected with 1×10^4^ PFU of the African ZIKV strain (ZIKV-AF) and found that such injections led to significant weight loss and premature death in treated mice by P6, compared with mock-treated mice (Fig. S1). This finding is consistent with the current literature that ZIKV-AF is a more virulent strain compared to others such as ZIKV-BR and causes more severe disease (Anfasa et al., 2017; Simonin et al., 2017; Tripathi et al., 2017). Nevertheless, we collected and measured the gross dimensions of brains from each treatment group at P3, and found that there was no significant difference in superficial length, superficial width and weight between infected (ZIKV-BR and ZIKV-AF) and control groups (Fig. S2A,C-E). We also performed measurements on P7 brains of ZIKV-BR and control groups and found that there was no significant difference in such measurements as well (Fig. S2B,F-H).

Following gross brain measurements, we investigated the effects of ZIKV on the SGZ of the DG within the hippocampus. This area starts to form at P7, and its structure is only clearly organized after P14 (Nicola et al., 2015). However, mice treated with 1×10^4^ PFU in our study did not survive to this age. Thus, we performed another set of injections to deliver a lower dose (30 PFU) of ZIKV-BR (hereafter denoted as ZIKV) per injection. In this revised model, we found that virus-infected mice significantly lost weight by P14, at which time their weight was documented to be less than half of that of control animals (Fig. 1C). Furthermore, we found that the brain weights of treated animals were significantly lower as well (Fig. S3).

Given that our ZIKV infection studies show an effect on brain weights in treated animals at P7 or older, we examined the effects between titers more closely. As shown, we observed a reduction in the length of the DG blades in P7 mice injected with 10^4 ^PFU of ZIKV, as well as in P14 mice injected with 30 PFU of ZIKV (Fig. 1L-R). Additionally, in control brains, cells were densely packed and organized as they formed the superior and inferior DG blades, but cells within infected brains were ectopically distributed and the borders of the blades were not clearly defined, resulting in blurring of the hilus and the SGZ structures. Furthermore, by performing and analyzing a three-dimensional reconstruction of the DG, we also observed a reduction in the area and volume of this structure in infected animals (Fig. 1F-K).

### ZIKV infection leads to alterations in apoptosis and cell proliferation, as well as tissue organization within the postnatal DG

**Fig. 2.**
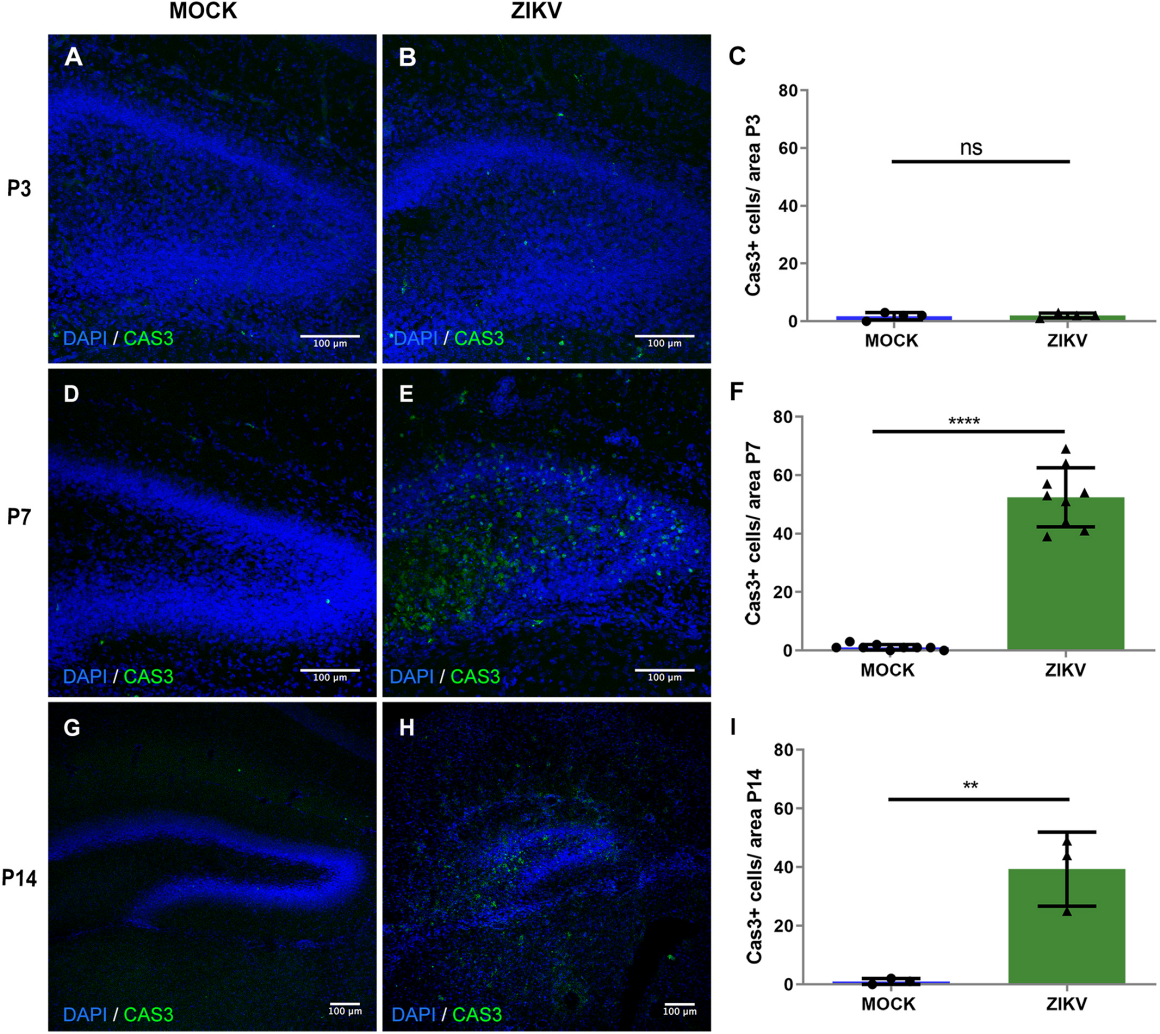
**ZIKV infection alters apoptosis within the postnatal hippocampal DG.** (A,B) Representative images of immunohistochemistry for activated caspase-3 (CAS3), a marker of apoptosis, in a DG coronal section of the mock and 10^4^ PFU ZIKV-BR groups at P3, counterstained with DAPI. (C) Quantification of CAS3^+^ cell density in the P3 DG. ns, not significant, *P*>0.05. *n*=8 (4 mock-treated and 4 ZIKV-injected animals). (D,E) Representative images of immunohistochemistry for CAS3 in a DG coronal section of the mock and 10^4^ PFU ZIKV-BR group at P7, counterstained with DAPI. (F) Quantification of CAS3^+^ cell density in the P7 DG. ****P<0.00001. *n*=18 (9 mock-treated and 9 ZIKV-injected animals). (G,H) Representative images of immunohistochemistry for CAS3 in a DG coronal section of the mock and 30 PFU ZIKV-BR groups at P14, counterstained with DAPI. (I) Quantification of CAS3^+^ cell density in the P14 DG. **P<0.001. *n*=6 (3 mock-treated and 3 ZIKV-injected animals). All data are presented as mean±s.e.m. Unpaired two-tailed Student's *t*-test was used for statistical analysis.

**Fig. 3.**
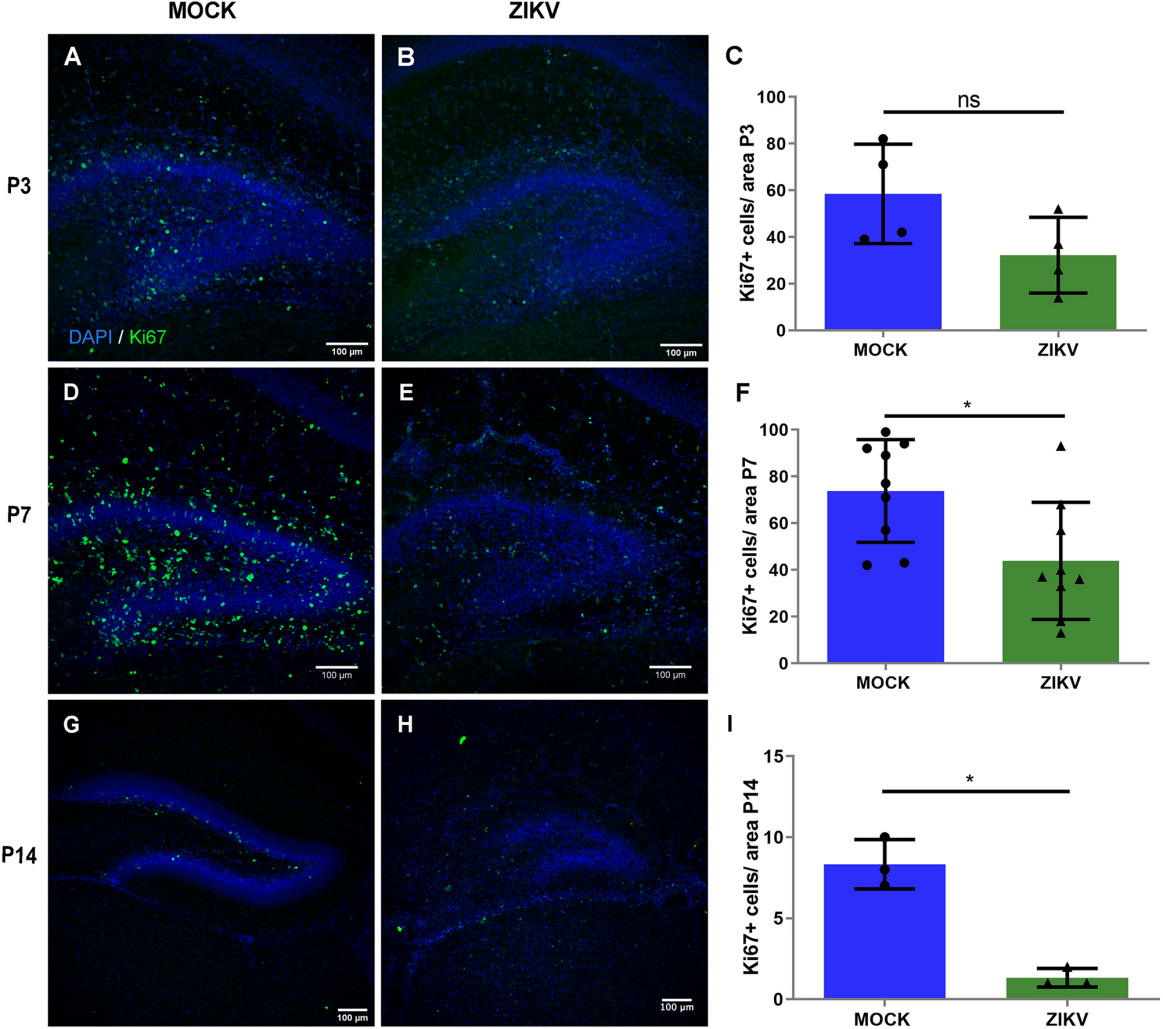
**ZIKV infection affects cell proliferation within the hippocampal DG.** (A,B) Representative images of immunohistochemistry for Ki67, a marker of cycling cells, in a DG coronal section of the mock and 10^4^ PFU ZIKV-BR groups at P3, counterstained with DAPI. (C) Quantification of Ki67^+^ cell density in the P3 DG. ns, not significant, *P*>0.05. *n*=8 (4 mock-treated and 4 ZIKV-injected animals). (D,E) Representative images of immunohistochemistry for Ki67 in a DG coronal section of the mock and 10^4^ PFU ZIKV-BR groups at P7, counterstained with DAPI. (F) Quantification of Ki67^+^ cell density in the P7 DG. *P<0.05. *n*=18 (9 mock-treated and 9 ZIKV-injected animals). (G,H) Representative images of immunohistochemistry for Ki67 in a DG coronal section of the mock and 30 PFU ZIKV-BR groups at P14, counterstained with DAPI. (I) Quantification of Ki67^+^ cell density in the P14 DG. *P<0.05. *n*=6 (3 mock-treated and 3 ZIKV-injected animals). All data are presented as mean±s.e.m. Unpaired two-tailed Student's *t*-test was used for statistical analysis.

The mouse hippocampal DG is a brain region characterized by a microenvironment that sustains neuronal proliferation throughout life, and its functions are pivotal to produce new granule cells that integrate into the hippocampal circuitry for essential functions, such as learning and memory (Bond et al., 2015). Considering the viral tropism for neural progenitor cells, we sought to determine whether ZIKV infections disrupted such cells during DG formation. We first evaluated the rate of apoptotic cell death in the DG, by cleaved caspase-3 (CAS3) staining (Fig. 2). As shown, the proportion of apoptotic cells between control mice and mice exposed to ZIKV at P0 and collected at P3 was not significantly different (Fig. 2A-C). However, the proportion of CAS3-positive (CAS3^+^) cells was significantly higher in mice infected at P0 and analyzed at P7, compared with that in control mice (Fig. 2D-F; Fig. S7). Similarly, the proportion of CAS3^+^ cells was significantly higher in mice infected with 30 PFU injections at P0 and analyzed at P14 (Fig. 2G-I). In addition to these observations, we quantified the proportion of cells stained with an anti-Ki67 antibody, a marker of cell proliferation (Fig. 3). As shown, the proportion of Ki67^+^ cells in control mice and in mice exposed to ZIKV at P0 and collected at P3 was not significantly different (Fig. 3A-C); however, the proportion of such cells was significantly lower in mice infected at P0 and analyzed at P7 (Fig. 3D-F) and in mice infected with 30 PFU injections at P0 and analyzed at P14 (Fig. 3G-I). Additionally, we found that in P7 infected mice, there was a lower proportion of Ki67^+^ cells that were positive for SOX2, a neural progenitor marker, and a higher proportion of Ki67+ cells that were co-localized with IBA1, a microglial marker (Fig. S6).

**Fig. 4.**
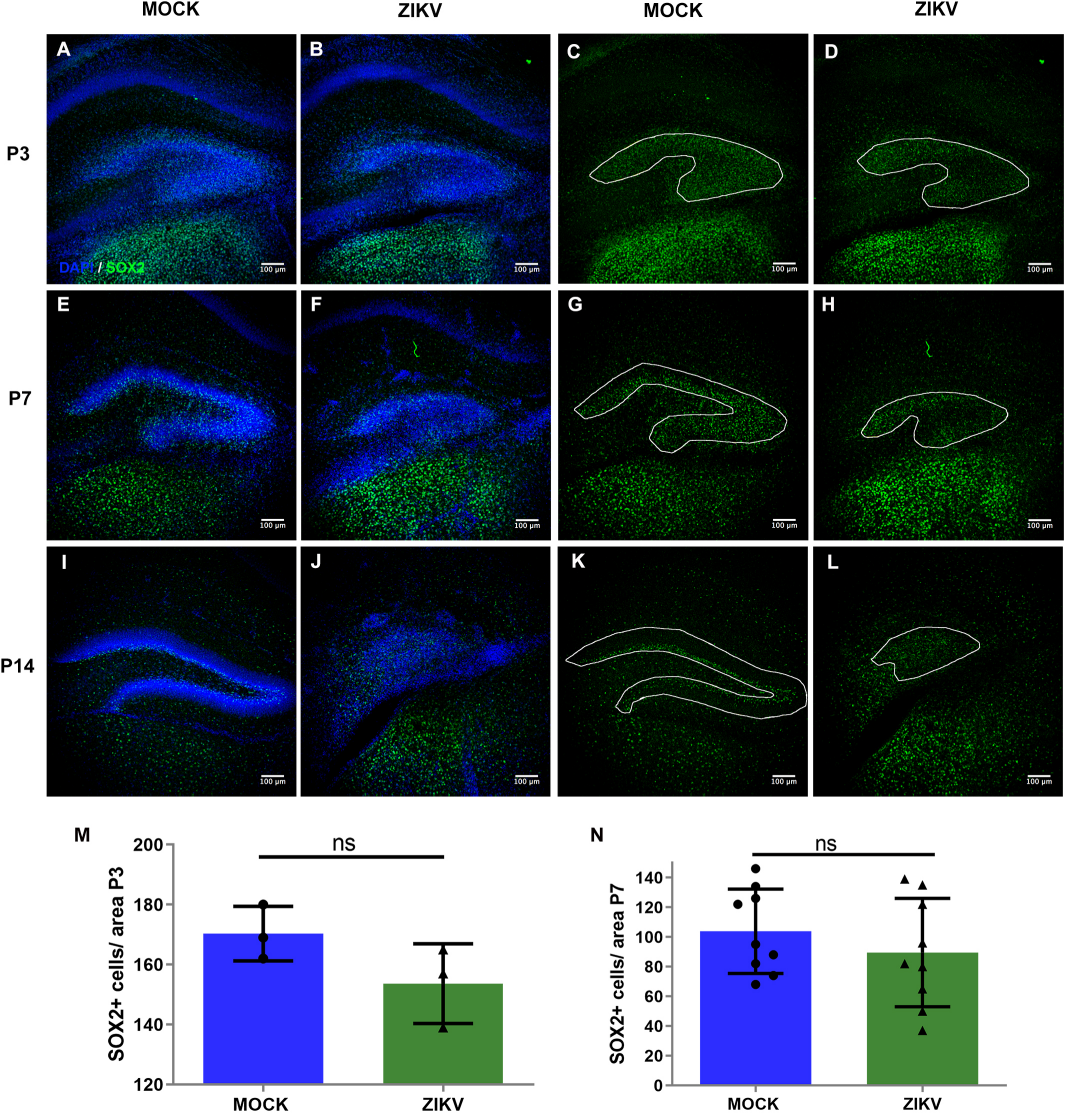
**ZIKV disrupts subgranular zone development and the positioning of SOX2^+^ cells.** (A-L) Representative images of immunohistochemistry for SOX2^+^ cells, markers of neural progenitors, in hippocampi from mock-treated or ZIKV-BR-injected (10^4^ PFU for analysis at P3 or P7, 30 PFU for analysis at P14) animals at P3 (A-D), P7 (E-H) and P14 (I-L), counterstained with DAPI, reveal an abnormal formation of the subgranular zone. The white lines show the borders of the granule cell layer. (M) Quantification of SOX2^+^ cell density in the P3 DG. *n*=6 (3 mock-treated and 3 ZIKV-injected animals). (N) Quantification of SOX2^+^ cell density in the P7 DG. *n*=18 (9 mock-treated and 9 ZIKV-injected animals). All data are presented as mean±s.e.m. Unpaired two-tailed Student's *t*-test was used for statistical analysis. ns, not significant, *P*>0.05.

During the formation of the neurogenic niche within the hippocampus, neural progenitor cells are redistributed to the SGZ of the DG, consistent with what we observed in the brains of control animals immunostained for SOX2 (Fig. 4). However, the equivalent, nascent neurogenic area within the brains of infected mice did not appear to develop as expected. Although we did not find differences in the number of SOX2^+^ cells between control and infected mice, SOX2-immunoreactive progenitor cells were distributed ectopically across the DG, instead of aligning within the subgranular region, as seen at P14 in control animals (Fig. 4).

### The cellular composition of the DG is affected by ZIKV infection

**Fig. 5.**
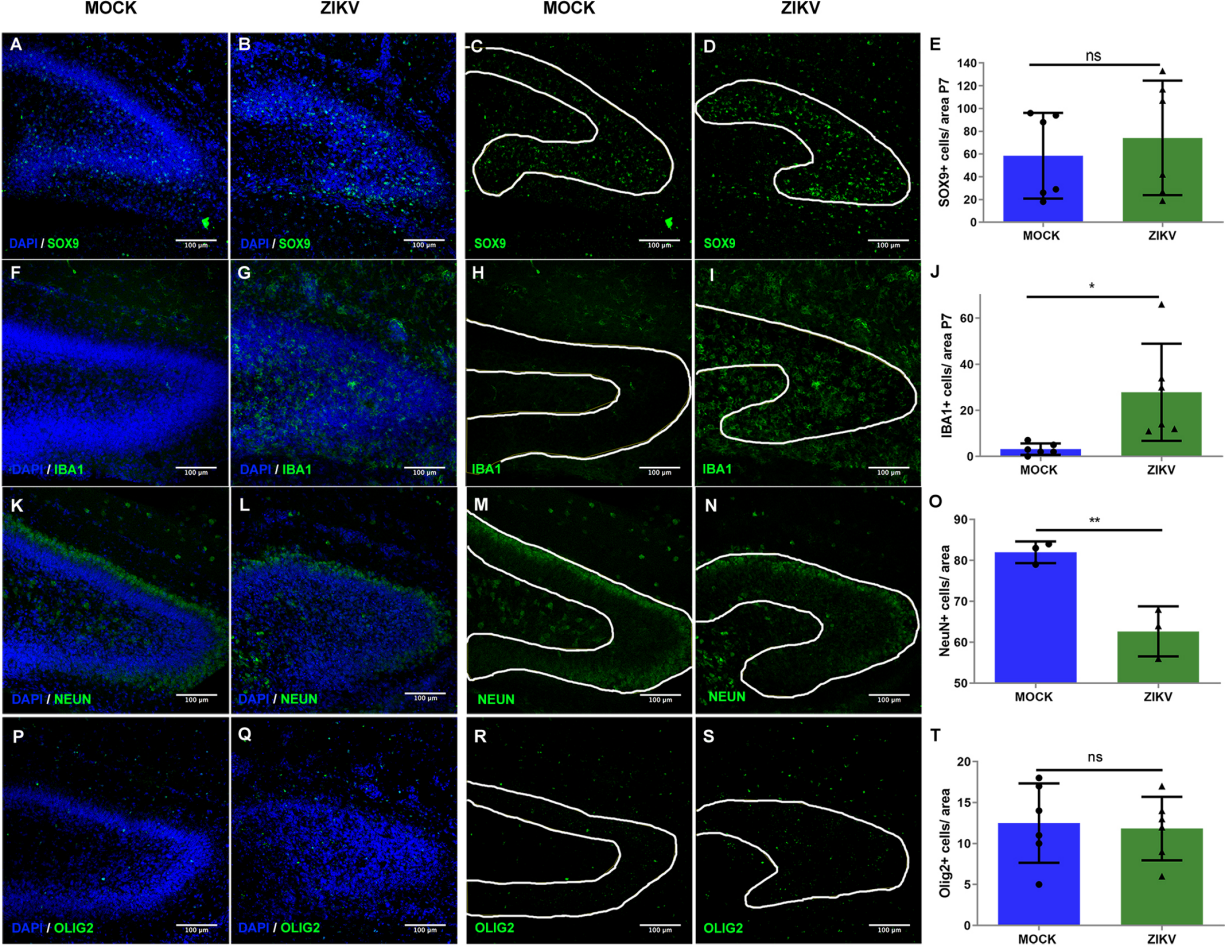
**ZIKV infection leads to abnormal cellular features within the P7 DG.** (A-D) Representative coronal sections of the DG from mock-treated and 10^4^ PFU ZIKV-BR-injected animals showing the distribution of SOX9^+^ cells. (E) Quantification of SOX9^+^ cell density. ns, non-significant, *P*>0.05. *n*=12 (6 mock-treated and 6 ZIKV-injected animals). (F-I) Representative coronal sections of the DG from mock-treated and 10^4^ PFU ZIKV-BR-injected animals showing the distribution of IBA1^+^ cells. (J) Quantification of IBA1^+^ cell density. *P<0.05. *n*=6 (6 mock-treated and 6 ZIKV-injected animals). (K-N) Representative coronal sections of the DG from mock-treated and 10^4^ PFU ZIKV-BR-injected animals showing the distribution of NEUN^+^ cells. (O) Quantification of NEUN^+^ cell density. **P<0.005. *n*=6 (3 mock-treated and 3 ZIKV-injected animals). (P-S) Representative coronal sections of the DG from mock-treated and 10^4^ PFU ZIKV-BR-injected animals showing the distribution of OLIG2^+^ cells. (T) Quantification of the OLIG2^+^ cell density. ns, not significant, *P*>0.05. *n*=12 (6 mock-treated and 6 ZIKV-injected animals). White lines mark the DG limits in the sections. All data are presented as mean±s.e.m. Unpaired two-tailed Student's *t*-test was used for statistical analysis.

Next, we performed a series of immunostainings to determine whether exposure to ZIKV influences cellular composition within the developing DG (Fig. 5). We found an increase in the number of microglia/macrophages identified by immunostaining for IBA1 (Fig. 5F-J) in virally infected pups compared with controls, but there were no differences in the number of astrocytes identified by SOX9 immunolabeling (Fig. 5A-E). However, we found an increased expression of GFAP in infected animals at P7, suggesting astroglial activation during ZIKV infection (Fig. S4A). Additionally, ZIKV infection increased expression of several pro-inflammatory cytokines such as IL-6, IL-1β (or IL1B) and TNFα (or TNF) at P7 and P14 and CX3CL1 at P14 (Fig. S4B-D). In addition to disruptions in these glial cell types, we also found a 25% reduction and abnormal distribution of NEUN (or RBFOX3)-immunostained neurons in the granular layer in the infected DG, which was statistically significant (Fig. 5O). Furthermore, although such neurons in control mouse brains are tightly packed in the granular layer, these cells were more loosely packed within ZIKV-infected mouse brains (Fig. 5K-N). In contrast, the relative frequency of oligodendrocytes marked by OLIG2 expression within the DGs was not statistically different (Fig. 5P-T).

**Fig. 6.**
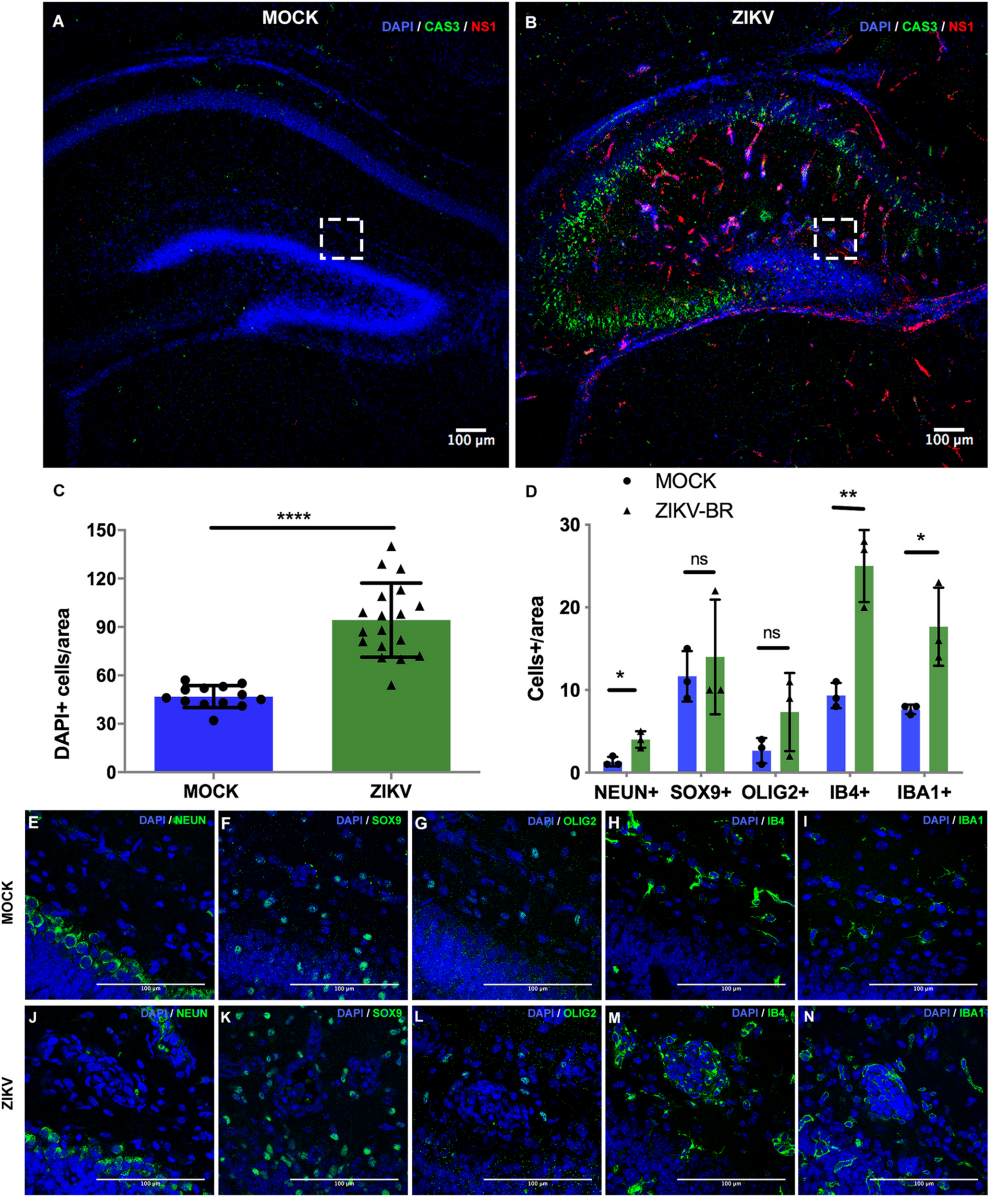
**ZIKV infection influences cellular composition within the hippocampal fissure region.** (A,B) Representative images of immunohistochemistry for ZIKV non-structural protein 1 (NS1) and CAS3 on the coronal section of the hippocampus at P7 from mock-treated and 10^4^ PFU ZIKV-BR-injected animals. (C) Quantification of DAPI^+^ cell density in the hippocampal fissure. ****P<0.00005. *n*=36 (18 mock-treated and 18 ZIKV-injected animals). (D) Quantification of NEUN^+^, SOX9^+^, OLIG2^+^, IB4^+^ and IBA1^+^ cell density in the hippocampal fissure from mock-treated and 10^4^ PFU ZIKV-BR-injected animals. ns, not significant, *P*>0.05; *P<0.05; **P<0.005. *n*=6 (3 mock-treated and 3 ZIKV-injected animals). All data are presented as mean±s.e.m. Unpaired two-tailed Student's *t*-test was used for statistical analysis. (E-N) High-magnification representative images of the NEUN^+^, SOX9^+^, OLIG2^+^, IB4^+^ and IBA1^+^ staining in the cell clusters at the hippocampal fissure from P7 mock-treated and 10^4^ PFU ZIKV-BR-injected animals. Dashed squares (A,B) represent the regions of the hippocampal fissure that were analyzed.

In addition to defects in the hippocampal DG of ZIKV-infected mice, we found abnormalities in the hippocampal fissure and adjacent regions. Particularly, we observed that the blood vessels stained with isolectin B4 (IB4) had different morphology. The control group presented long and ramified vessels, and, although the infected animals had 57.5% more blood vessels, these were 26% shorter and 26.5% less branched than control blood vessels (Fig. S5). We also found heterotopic clusters of cells above the molecular layer within the hippocampal fissure of infected animals at P7 (Fig. 6A,B). Within these clusters, there were approximately twice the number of cells found in the hippocampal fissure of control mouse brains (Fig. 6C). Furthermore, 29.03% of these cells were CAS3^+^ and 5.8% overlapped with NS1 immunostaining, which marks ZIKV-infected cells. This agglomerated cell pattern above the DG has not been described in previous studies with ZIKV infection. To investigate in detail the identity of these cells and analyze possible changes in the cellular composition of the hippocampal fissure, we quantified various cell types by immunostaining and found significant increases in IBA1^+^ microglial cells, IB4^+^ endothelial cells, as well as NEUN^+^ neurons in virally infected brains compared to those in control. There were no significant differences in the relative proportions of SOX9^+^ astrocytes and OLIG2^+^ oligodendrocytes within this region (Fig. 6D-N). Taken together, our model of intraventricular ZIKV infection in neonatal (P0) mice induced significant cellular changes and development effects within the postnatal (P7) DG and hippocampal fissure, but without changes in gross brain measurements.

## DISCUSSION

This study characterizes a new CZS model for the study of hippocampal development. We show that ZIKV alters the morphology, cell composition and cell distribution during the development of the DG in wild-type C57BL/6 mice that were infected intracranially in the early postnatal period (P0).

**Fig. 7.**
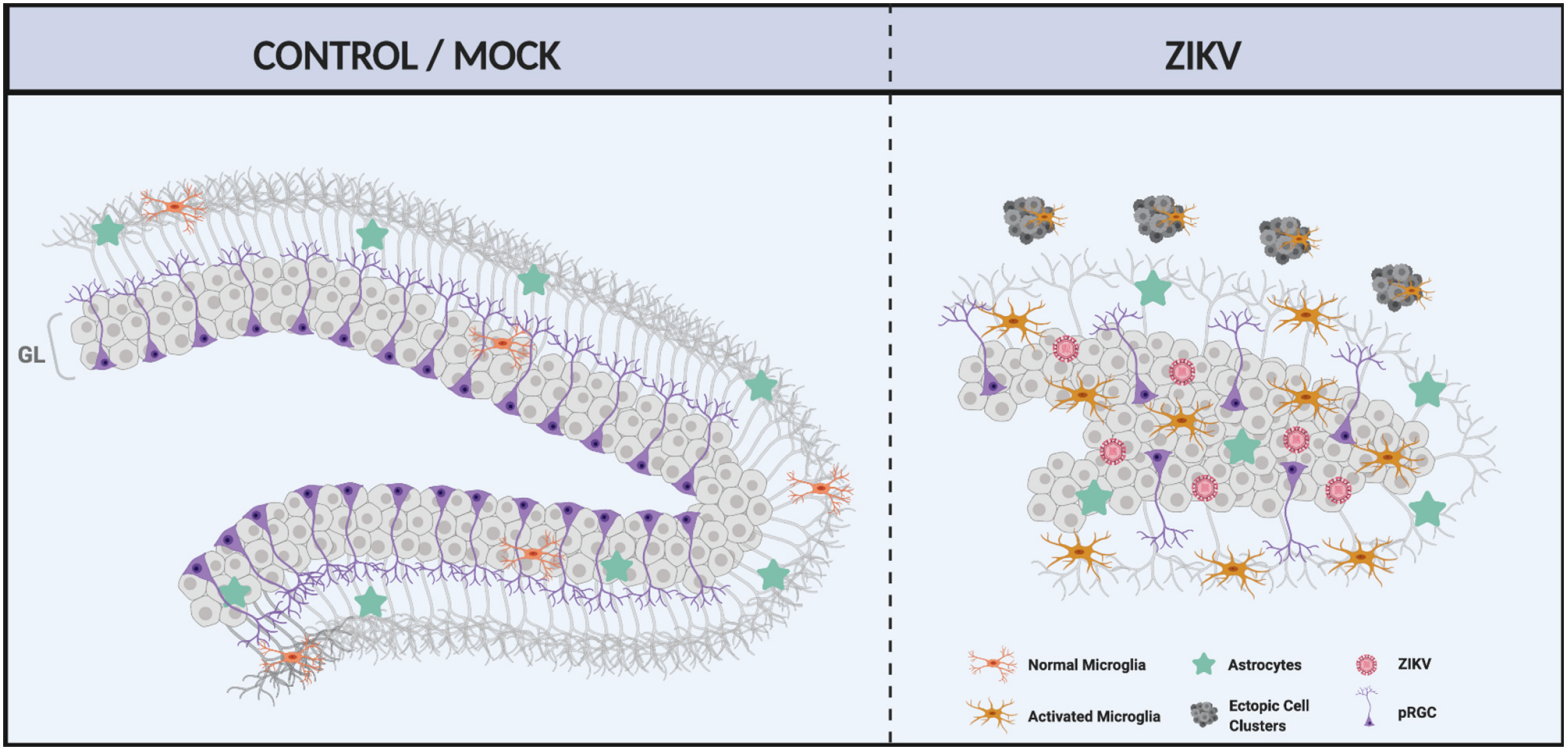
**Summary of the effects of neonatal (P0) ZIKV infection on postnatal DG development.** Left: illustration of the typical DG, showing the distinct layers and characteristic shape of this structure. Right: representation of the ZIKV-infected DG. Besides being smaller, the DG has a vast disorganization of the subgranular zone and altered shape. Moreover, the cellular composition of the ZIKV-infected DG is altered. There is a reduction in the number of neurons and an increased number of microglial cells and astrocytes. At the hippocampal fissure level, the ZIKV-infected animals show ectopic microglial/neuronal clusters that could be a result of DG cell detachment. GL, granular layer; pRGC, progenitor radial glial cells. Image created with BioRender.com.

In the postnatal mouse brain, neurogenesis is an essential and highly coordinated process that is regulated by intrinsic and extrinsic factors that influence zones of active proliferation, such as the hippocampal SGZ. Indeed, postnatal neurogenesis is relevant to brain homeostasis, aging and neurological disease (Bowers and Jessberger, 2016), with studies describing an association between age-dependent health conditions and reductions in NSC proliferation within the cortical and hippocampal SGZ, accompanied by phenotypes including cell dimorphism, disorganized cell layers and loss of granule cells of the DG (Adams Waldorf et al., 2018; Büttner et al., 2019; Li et al., 2016; Moreno-Jiménez et al., 2019). In this study, we found that ZIKV infection reduces DG area and volume (Fig. 7). Such alterations are often associated with neuropsychiatric conditions including post-traumatic stress disorder, schizophrenia and major depressive disorder (HAYES et al., 2017; Nakahara et al., 2020; Roddy et al., 2019). Additionally, major depressive disorder is also characterized by disorganized granular cells, reminiscent of what we found in our study, where the neurons were ectopically dispersed in the DG instead of being concentrated in the granular layer (Houser, 1990). As such, our findings may have implications for children with CZV featuring dysmorphic cortical SVZ and hippocampal DG features, which influence their susceptibility to present with such neuropsychiatric disorders later in life.

The disorganization of hippocampal cells within the SGZ is found in Reeler mice, wherein such defects are attributable to the lack of expression of reelin (RELN), a protein synthesized and secreted by the Cajal–Retzius cells in the hippocampal fissure extracellular matrix to promote neuronal migration (Freiman et al., 2011; Haas et al., 2002; Heinrich et al., 2006). Given this mechanism for cell positioning within the developing hippocampus, we speculate that cellular imbalance resulting from ZIKV infection-induced cellular changes within the hippocampal fissure (Fig. 6) disrupts reelin signaling.

Previous studies have shown that ZIKV infection leads to a relative reduction in neurons within the DG (Bell et al., 1971; Büttner et al., 2019; Li et al., 2018). Consistent with this finding, we find in our model that ZIKV infection leads to increased cell apoptosis and reduced numbers of NEUN^+^ cells. Additionally, our model for ZIKV infection also features disorganization of SOX2^+^ cells and reduced numbers of proliferating cells immunolabelled with Ki67, suggesting possible impairment in the pool of progenitors that exacerbates defective neurodevelopment, altogether resulting in reduced hippocampal tissue growth and maturation.

We found that ZIKV infection can alter DG cellularity. Additionally, we observed an increased GFAP expression in the hippocampus of infected animals, indicating astrocyte reactivity. In addition, pro-inflammatory mediators such as cytokines may induce microglial activation. In our infection model, we found several pro-inflammatory cytokines with increased expression in the hippocampus. These results suggest that neuroinflammation in the hippocampus is mediated by astrogliosis and microglia activation. Other studies have also shown astrogliosis and microglial activation as a result of ZIKV infection (Büttner et al., 2019; Shao et al., 2016). This cellularity change found in the DG can alter the normal function of this region as reactive astrocytes and microglial activation could negatively impact neurogenesis (Wang et al., 2018).

Here, we show that ZIKV impairs the development and the organization of the DG neurogenic area in a murine model of postnatal infection. One limitation of our study is that, by delivering the virus directly to the mouse brains, we exclude an important factor of initial ZIKV pathogenesis, which is the participation of the peripheral immune system. However, an intracerebroventricular injection allowed us to study the direct effects of ZIKV infection on the brain and led to neuropathological changes that have been observed in other animal models and the syndrome (Cui et al., 2017; Nem de Oliveira Souza et al., 2018). In addition, infected animals had a high mortality rate, even when the viral titer was reduced, thus limiting the analysis of brain development in later stages until adulthood.

However, by describing the alterations in cellularity and architecture of the DG after ZIKV infection, this study contributes to a better characterization of the abnormal developmental mechanisms found in the CZS and helps in understanding the cognitive impairments of affected newborns during their development until they reach adulthood.

## MATERIALS AND METHODS

### Animals and infection

All experimental procedures were performed in accordance with the Brazilian National Council for the Control of Animal Experimentation (CONCEA) guidelines and were approved by an Institutional Ethics Committee (protocol CEUA-CCS/UFRJ A18/19-040-19). C57BL/6 mice were obtained from the Animal Care Facility of the Microbiology Institute of the Federal University of Rio de Janeiro. Animals were kept under a 12 h:12 h light-dark cycle in a temperature-controlled environment at 23°C. Mice had *ad libitum* access to food and water.

Neonatal mice were divided into groups and injected on both lateral cerebral ventricles at P0 with 1×10^4^ PFU of the Brazilian ZIKV strain (ZIKV-BR; Recife/Brazil, ZIKV PE/243, number KX197192.1), the African ZIKV strain (ZIKV-AF; MR766 – Uganda/Africa, number NC012532.1) or a mock solution {supernatant of Vero cells [American Type Culture Collection (ATCC), CCL-81] or C6/36 cells (kindly gifted by Dr Claire Kubelka, Fundação Oswaldo Cruz)} after anesthesia induced by hypothermia. Neonatal mice harvested at P14 were injected with 30 PFU ZIKV at P0. ZIKV strains were provided by the Laboratory of Molecular Virology at the Institute of Biology, Department of Genetics, Federal University of Rio de Janeiro. ZIKV titers were determined by plaque assay.

### Euthanasia and tissue preparation

Mice were euthanized on P3, P7 and P14. Due to the high lethality of the ZIKV-AF, the analyses were only performed at P3. Animals were weighed and photographed prior to being euthanized. Mice were anesthetized with ketamine (120 mg/kg) and xylazine (12 mg/kg) and perfused with cold PBS (0.1 M) followed by 4% paraformaldehyde. For PCR and plaque assays, mice were euthanized by decapitation and the whole brain or hippocampus was collected in dry ice and stored at −80°C.

### Assessment of body weight, brain size and brain weight

To characterize the effects of the ZIKV infection, we measured the weights of mice from the respective treatment groups and the brain weights at different time points. Images of the dorsal view of the brains were taken with an optical microscope (Leica, DFC490); *n*=12 litters (five mock, four ZIKV-BR-infected and three ZIKV-AF-infected brains). The lengths (rostro-caudal) and widths (latero-medial) of the brains were measured (in micrometers) using ImageJ software.

### Immunohistochemistry

Coronal sections (80 μm) were obtained with a vibratome (Leica, Germany), permeabilized with 0.2% Triton X-100 (Sigma-Aldrich) and incubated with 3% bovine serum albumin (Sigma-Aldrich). After standard antigenic retrieval using sodium citrate, the free-floating sections were incubated overnight with the following primary antibodies: anti-Caspase-3 (1:300, Millipore, AB3623); rabbit anti-Ki67 (1:100, Millipore, AB9260); mouse anti-NS1 (1:10, Thermo Fisher Scientific, MA5-24585); rabbit anti-SOX2 (1:200, Sigma-Aldrich/Millipore, AB5603); rabbit anti-SOX9 (1:200, Abcam, ab185230); mouse anti-IBA1 (1:1000, Millipore, MABN92); mouse anti-NEUN (1:200, Millipore, MABN377); and rabbit anti-OLIG2 (1:300, Millipore, MABN50). Following the overnight incubation, sections were washed with PBS and incubated for 2 h with goat anti-rabbit Alexa Fluor 488 (1:500, Millipore, AP132JA4) and goat anti-mouse Alexa Fluor 546 (1:500, Invitrogen, A11003) secondary antibodies. The free-floating sections that were stained with isolectin B4 (IB4; 1:200, Invitrogen, 121411) were incubated with the stain together with the secondary antibodies. Nuclei were stained with DAPI (0.5 μg/ml) for 30 min. Sections were placed in glass slides and mounted with Fluoromount (Sigma-Aldrich, F4680).

### Tridimensional cortical reconstruction

Perfused and fixed P7 brains were used to perform tridimensional reconstruction of relevant structures. Coronal sections (150 μm) were obtained with a vibratome (Leica, Germany). The first 13 sections after the appearance of midline-crossing callosal fibers were selected and mounted serially into poly-L-lysine (Sigma-Aldrich)-coated glass slides. The sections were submitted through a Nissl staining protocol in which they were stained with Cresyl Violet for 5 min, dehydrated with a sequence of ethanol solutions that were increasingly concentrated and clarified with xylene. The slides were sealed with Entellan Mounting Medium (Merck).

With the bright field of an Eclipse 80i (Nikon) Microscope associated with the Neurolucida Software (MBF Biosciences), we traced the contours of the DG and of the external borders of each slice. From the contours that were aligned in sequence and the slice thickness measurement (150 µm), the volume measurements were extrapolated and extracted with the Neurolucida Explorer Software (MBF Biosciences). To adjust for variations between individuals, we divided the values of the areas and volumes of the DG by the areas or volumes of its respective external contour.

### Imaging, quantification and statistical analysis

Images of three immunostained sections per animal were acquired with a laser scanning confocal microscope (Leica, DFC500) with 10×, 20×, 40× or 63× objective lenses. The images with 63× magnification for cell quantification were acquired in the DG crest region (intersection of the superior and inferior blades), and in the superior blade, results were averaged between the two regions. ImageJ was used to count the immunoreactive cells and to measure the length of the DG blades. For blood vessel analysis, images with 10× magnification were acquired with a laser scanning confocal microscope (Leica, DFC500) and analyzed using AngioTool (Zudaire et al., 2011). Unpaired two-tailed Student's *t*-test or one-way ANOVA with Tukey's test for multiple comparisons was performed using GraphPad Prism 6.0. Data in graphs are presented as mean±s.e.m. *P≤0.05, **P≤0.01, ***P≤0.001, ****P≤0.0001.

### Viral detection and quantification

P3 and P7 brains were collected for viral detection. The samples were weighed and kept at −80°C until processing. Plaque assay was performed for viral quantification.

#### Cell lines

Vero (ATCC, CCL-81) and Vero E6 (ATCC, CRL-1586) cell lines were maintained in Dulbecco's modified Eagle medium (DMEM; Gibco) supplemented with 5% (v/v) fetal bovine serum (FBS; Gibco) at 37°C and 5% CO_2_.

#### Tissue maceration

Samples were diluted in 1 m; DMEM supplemented with 2% (v/v) FBS and 2% (v/v) antibiotics and macerated with a T 10 basic Ultra-Turrax (IKA) dispersing instrument. Following 5 min of centrifugation at 3400 ***g***, the supernatant was harvested and stored at −80°C. Between each sample, the Ultra-Turrax was washed with PBS, 70% ethanol and Milli-Q sterile water two times each.

#### Virus titration

Virus titration was performed by plaque assay in Vero cells plated at a density of 6×10^4^ cells/well in 12-well plates 2 days prior to infection. After 1 h incubation with 200 μl of 10-fold serially diluted samples, the medium was replenished with minimum essential medium (MEM; Gibco) supplemented with 2% (v/v) FBS, 2% (v/v) antibiotics and 1.8% (v/v) carboxymethyl cellulose (CMC) (Sigma-Aldrich), and incubated at 37°C and 5% CO_2_ for 5 days. Cells were fixed with 10% formaldehyde for 30 min at room temperature and stained with 20% (v/v) ethanol-Violet Crystal solution for 1 min. The viral titers were presented as plaque forming units (PFUs) per milliliter and were normalized by the weight of the sample (in grams).

### mRNA expression

Gene expression (Table S1) was measured in the mouse cerebral tissue (P7 and P13). Total RNA was isolated using TRIzol (Life Technologies). 1 µg of total RNA from the hippocampus was reverse transcribed using the High-Capacity cDNA Reverse Transcription Kit (Thermo Fisher Scientific) as suggested by the manufacturer. The reaction product was amplified by real-time PCR (QuantStudioTM 7 Flex Real-Time PCR, Life Technologies) using 10 ng cDNA and primers (600 nM) for the SYBR Green reaction (Thermo Fisher Scientific). For data analysis, the quantitative values for the expression were obtained from the 2^ddCt^ parameter, in which dCt represents the subtraction of the Ct values of the reference gene from those of the target gene and ddCt the normalization of the samples to the sample group mean. The final data are expressed as the ratio of the relative expression (fold change) of the alteration in the target gene of the experimental groups (ZIKV) over the variation in the target gene of the control groups (mock). For better analysis and visualization of fold changes in the data obtained, the graphs are presented using logarithmic base 2. The primers used for real-time PCR are given in Table S1.

## Supplementary Material

10.1242/dmm.050375_sup1 Supplementary information
