# Supplementary material for: Postnatal Zika virus infection leads to morphological and cellular alterations within the neurogenic niche
Source: Dis Model Mech. 2024 Feb 28;17(2):dmm050375. doi: 10.1242/dmm.050375 (PMC10924234; doi:10.1242/dmm.050375)
Supplement: Supplementary information [file dmm-17-050375-s1.pdf]

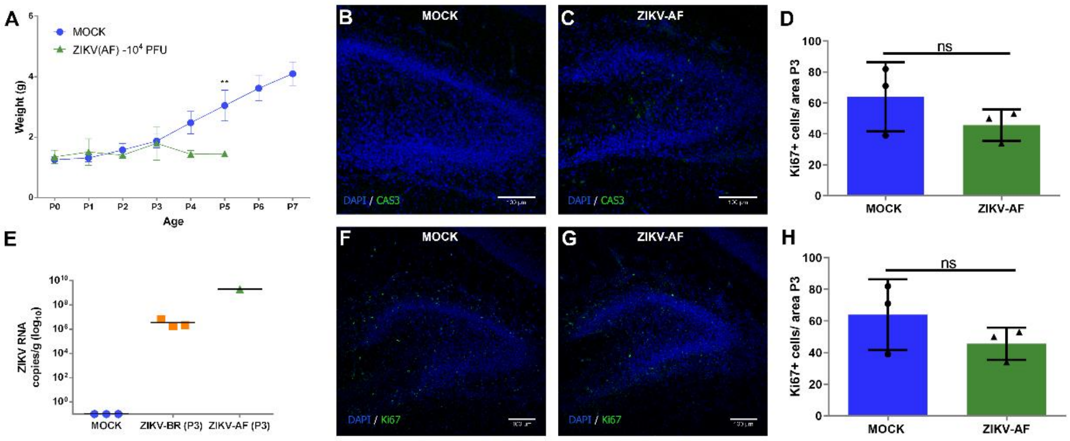

Fig. S1. Impact of ZIKV-AF on the dentate gyrus at P3.

(A) Weight gain (g) in the first seven days post 10^4 PFU intracranial injection ZIKV-AF. Data presented as mean ± SEM, n= 40; 20 MOCK animals and 20 ZIKV-AF animal from 6 different litters, Student's *t*-test **p<0.005.

(B-C) Representative images of the immunohistochemistry for activated caspase-3 (CASP3), a marker of apoptosis, in a dentate gyrus coronal section of the MOCK and ZIKV-AF groups at P3, counterstained with DAPI.

(D) Quantification of CASP+ cell density in the P3 dentate gyrus. Student's *t*-test, *p<0.05; n=6: 3 MOCK e 3 ZIKV-AF.

(E) Viral load measured by plaque brain assay in Plaque Forming units per gram (PFU/g). n=7: 3 MOCK, 3 ZIKV-BR and ZIKV-AF, P3.

(F-G) Representative images of the immunohistochemistry for Ki67, a marker of cycling cells, in a dentate gyrus coronal section of the MOCK and ZIKV-AF groups at P3, counterstained with DAPI.

(H) Quantification of Ki67+ cell density in the P3 dentate gyrus. Student's *t*-test, p> 0.05; n=6: 3 MOCK and 3 ZIKV. ns: non-significant.

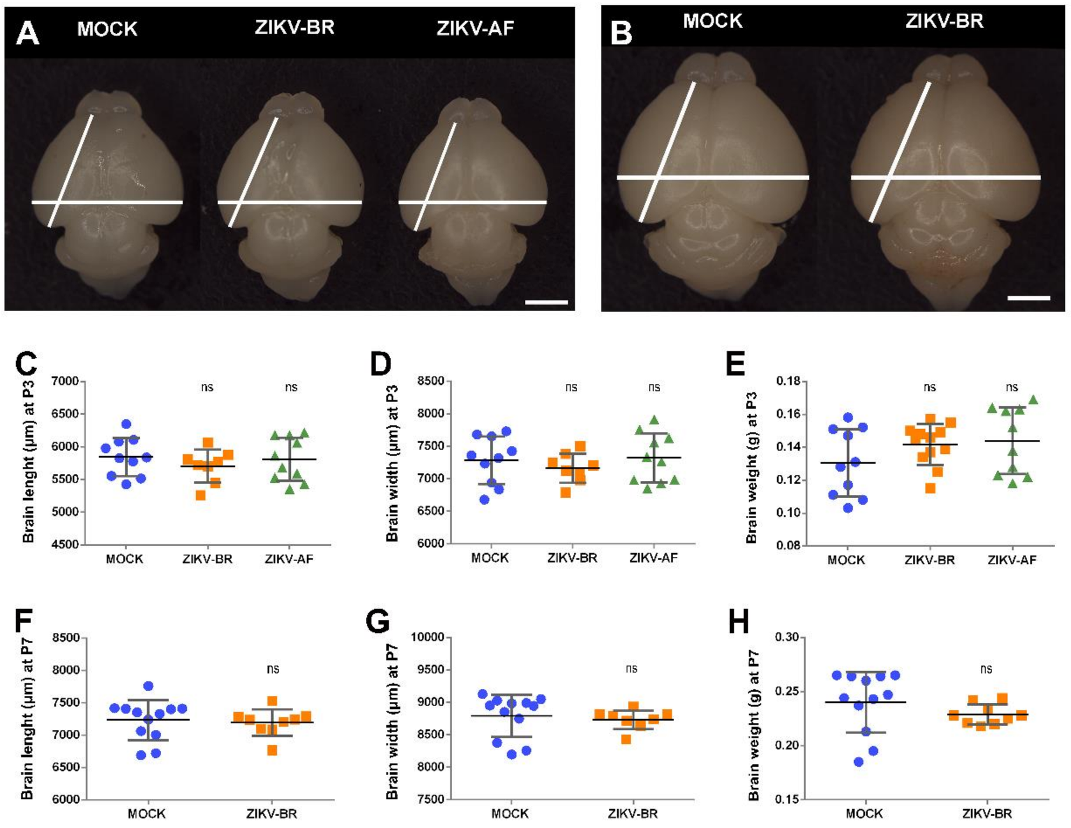

Fig. S2. Brain measurements (length, width, and weight) of mice infected with ZIKV. (A) MOCK, ZIKV-BR and ZIKV-AF 10^4 PFU brains at P3, respectively. Horizontal lines represent brain width and diagonal lines represent brain length. Scale bar represents 2mm.
(B) MOCK and ZIKV-BR 10^4 PFU brains at P7, respectively. Horizontal lines represent brain width and diagonal lines represent brain length. Scale bar represents 2mm.
(C-E) Quantification of the brain measurements at P3: length, width and weight, respectively; one-way ANOVA, $p > 0.05$; ns non-significant; n=28: 10 MOCK, 8 ZIKV-BR e 10 ZIKV-AF;
(F-H) Quantification of the brain measurements at P7: length, width and weight, respectively; Student's *t*-test, $p > 0.05$; ns: non-significant.; n=21: 12 MOCK and 9 ZIKVBR 10^4 PFU.

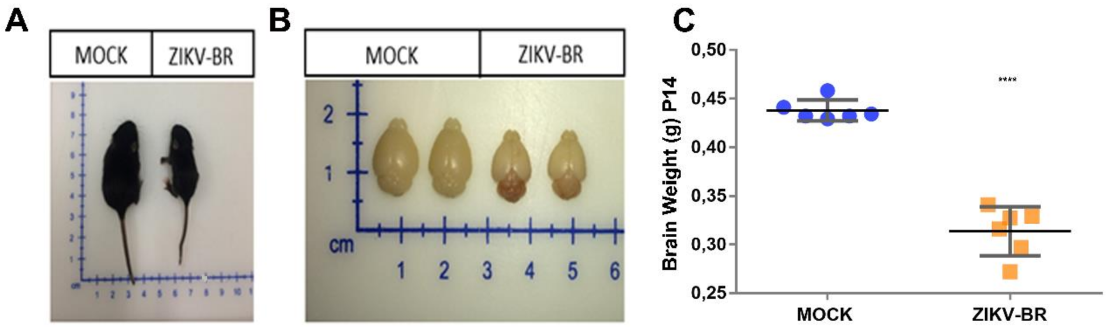

Fig. S3. ZIKV infection impacts on the dentate gyrus at P14.

(A) Experimental mice at P14. MOCK on the left and ZIKV-BR 30 PFU on the right.

(B) Brains at P14. MOCK on the left and ZIKV-BR 30 PFU on the right.

(C) Graphical representation of the brain weights at P14; Student's t-test.

****p<0.00001, n=12: 6 MOCK and 6 ZIKV-BR 30 PFU.

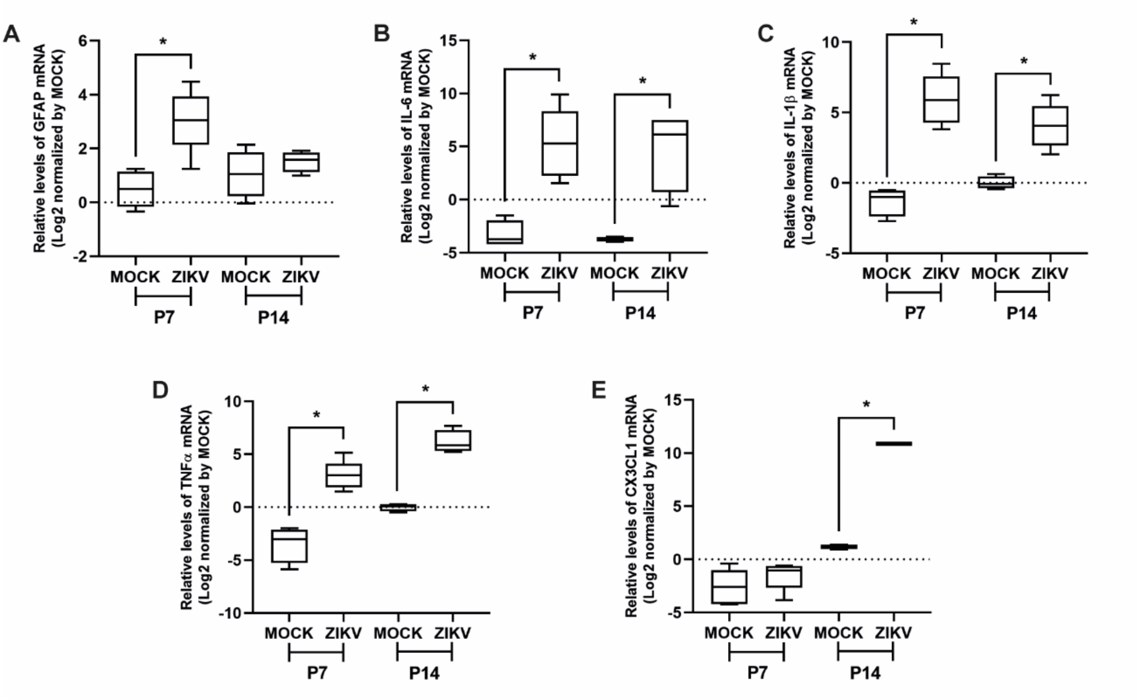

Fig. S4. Postnatal ZIKV infection induces neuroinflammation in mice hippocampus. Hippocampus mRNA expression of neuroinflammatory markers of (A) GFAP (B) IL-6 IL-1β (D) TNFα and (E) CX3CL1 in MOCK and ZIKV-BR infected animals at P0 and collected at P7 and P14. N= 24; 6 MOCK P7, 6 ZIKV-BR 10^4 PFU P7, 6 MOCK P14 and 6 ZIKV 30 PFU P14.

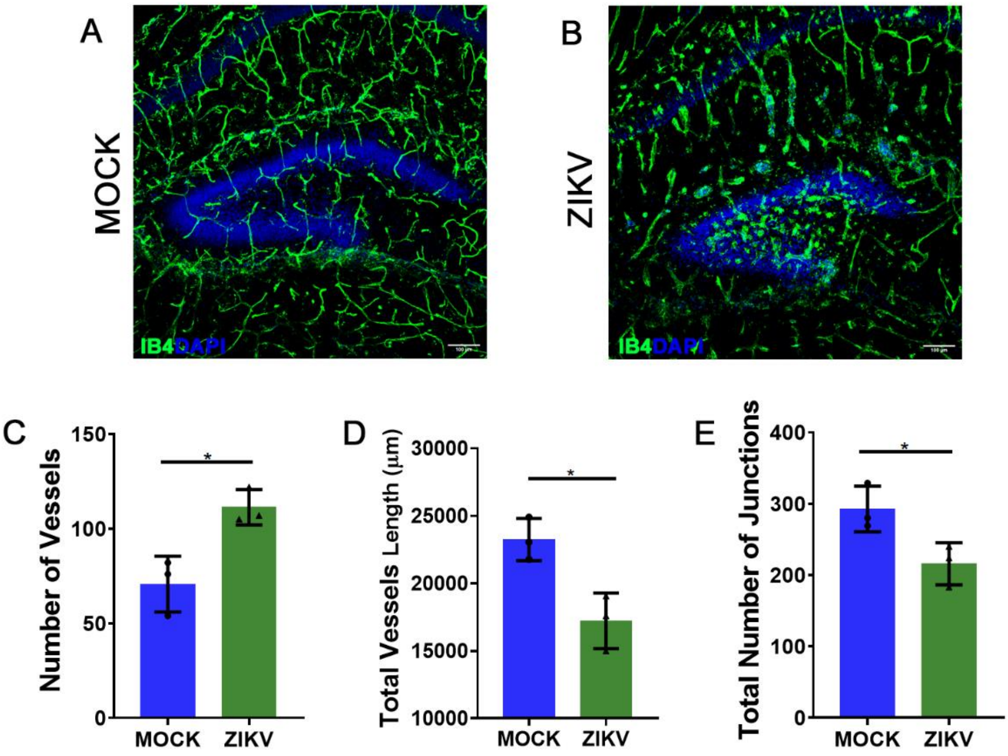

Fig. S5. ZIKV disrupts blood vessel organization in the hippocampus at P7.

(A, B) Representative images of the Immunohistochemistry for IB4 (Isolectin 4) in hippocampal coronal sections of the MOCK and ZIKV-BR 10^4 PFU groups at P7, counterstained with DAPI. Scale bar = 500 µm.

(C) Graphical representation of the number of blood vessels at P7; Student's t-test. *p<0,05. n= 6: 3 MOCK and 3 ZIKV-BR 10^4 PFU.

(D) Graphical representation of the total length of blood vessels at P7; Student's t-test. *p<0,05. n= 6: 3 MOCK and 3 ZIKV-BR 10^4 PFU.

(E) Graphical representation of the total number of junctions at P7; Student's t-test. *p<0,05. n=6: 3 MOCK and 3 ZIKV-BR 10^4 PFU.

Disease Models & Mechanisms • Supplementary information

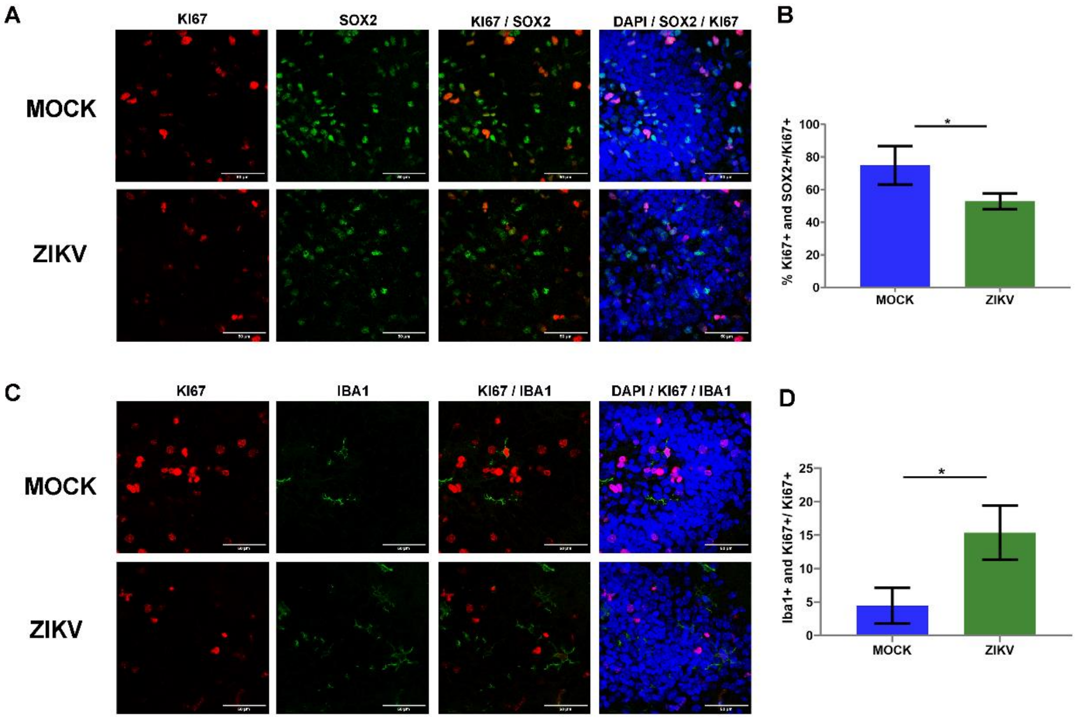

Fig. S6. Zika Virus infection alters the expression pattern of proliferating cells in the dentate gyrus.

(A) Representative images of the immunohistochemistry for Ki67 and SOX2 in a dentate gyrus coronal section of the MOCK and ZIKV-BR 10^4 PFU group at P7, counterstained with DAPI.

(B) Quantification of Ki67+/SOX2+ cell density in the P7 dentate gyrus. Student's *t*-test, *p> 0.05; n=6: 3 MOCK, 3 ZIKV-BR 10^4 PFU.

(C) Representative images of the immunohistochemistry for Ki67 and IBA1 in a dentate gyrus coronal section of the MOCK and ZIKV-BR 10^4 PFU group at P7, counterstained with DAP.

(D) Quantification of Ki67+/IBA+ cell density in the P7 dentate gyrus. Student's *t*-test, *p> 0.05; n=6: 3 MOCK, 3 ZIKV-BR 10^4 PFU.

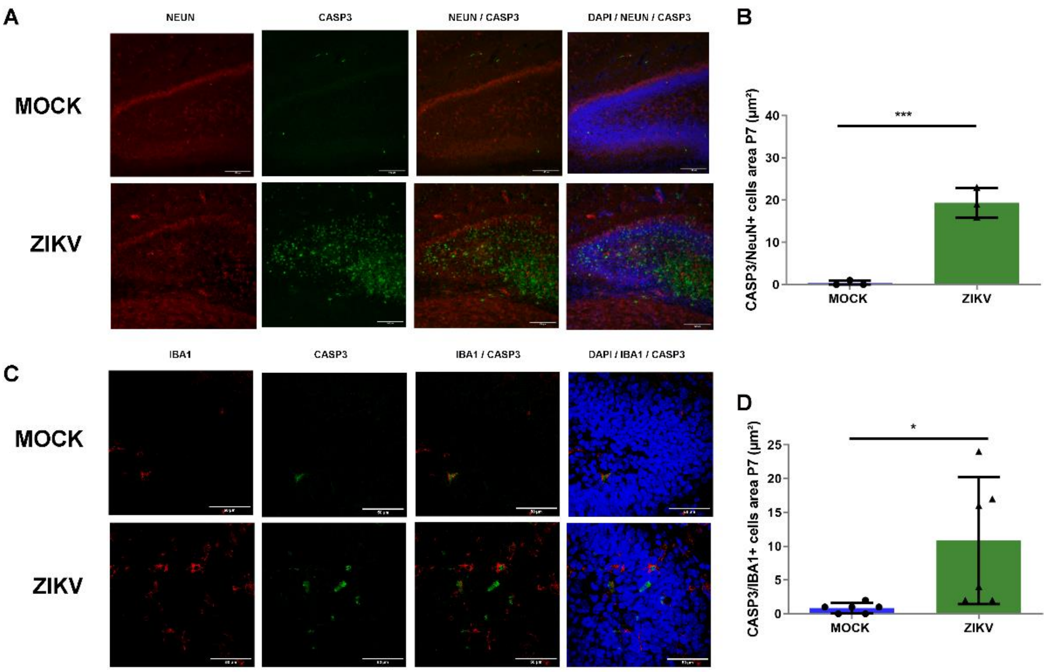

Fig. S7. Zika virus infection alters apoptosis of NEUN and IBA1 within the postnatal hippocampal dentate gyrus.

(A) Representative images of the immunohistochemistry for activated caspase-3 (CASP3), a marker of apoptosis and NEUN, in a dentate gyrus coronal section of the MOCK and ZIKV-BR 10^4 PFU group at P7, counterstained with DAPI.

(B) Quantification of CASP3/NEUN+ cell density in the P7 dentate gyrus. Student's t-test. ***p<0.0001, n=6: 3 MOCK and 3 ZIKV-BR10^4 PFU.

(C) Representative images of the immunohistochemistry for CASP3/IBA1+ in a dentate gyrus coronal section of the MOCK and ZIKV-BR 10^4 PFU group at P7, counterstained with DAPI.

(D) Quantification of CASP3/IBA1+ cell density in the P7 dentate gyrus. Student's *t*-test, *p<0.05. n=12: 6 MOCK, 6 ZIKV-BR 10^4 PFU.

Table S1. Target genes and primers sequence to detect mouse mRNA

Target Gene	Specificity	Sequence
GFAP	Mouse	F: GCC ACC AGT AAC ATG CAA GA<br>R: GCT CTA GGG ACT CGT TCG TG
IL-6	Mouse	F: TTC TTG GGA CTG ATG CTG GTG<br>R: CAG AAT TGC CAT TGC ACA ACT C
IL-1ß	Mouse	F: GAT ATG AAA GAC GGC ACA CC<br>R: ATT AGA AAC AGT CCA GCC CA
TNFα	Mouse	F: CCC TCA CAC TCA GAT CAT CTT CT<br>R: GCT ACG ACG TGG GCT ACA G
CX3CL1	Mouse	F: GGC TAA GCC TCA GAG CAT TG<br>R: CTG TAG TGG AGG GGG ACT CA

Disease Models & Mechanisms • Supplementary information
